# Clinical, humanistic, and economic burden of severe hemophilia B in the United States: Results from the CHESS US and CHESS US+ population surveys

**DOI:** 10.1186/s13023-021-01774-9

**Published:** 2021-03-20

**Authors:** Tom Burke, Sohaib Asghar, Jamie O’Hara, Eileen K. Sawyer, Nanxin Li

**Affiliations:** 1 HCD Economics, Daresbury, UK; 2 Faculty of Health and Social Care, University of Chester, Chester, UK; 3 uniQure Inc, 113 Hartwell Avenue, Lexington, MA 02421 USA

**Keywords:** Hemophilia B, Factor IX, Burden, Cost, Bleeds, Health-related quality of life, Patient-reported outcomes, Real-world

## Abstract

**Background:**

Hemophilia B is a rare congenital bleeding disorder that has a significant negative impact on patients’ functionality and health-related quality of life. The standard of care for severe hemophilia B in the United States is prophylactic factor IX replacement therapy, which incurs substantial costs for this lifelong condition. Accurate estimates of the burden of hemophilia B are important for population health management and policy decisions, but have only recently accounted for current management strategies. The ‘Cost of Severe Hemophilia across the US: a Socioeconomic Survey’ (CHESS US) is a cross-sectional database of medical record abstractions and physician-reported information, completed by hematologists and care providers. CHESS US+ is a complementary database of completed questionnaires from patients with hemophilia. Together, CHESS US and CHESS US+ provide contemporary, comprehensive information on the burden of severe hemophilia from the provider and patient perspectives. We used the CHESS US and CHESS US+ data to analyze the clinical, humanistic, and economic burden of hemophilia B for patients treated with factor IX prophylaxis between 2017 and 2019 in the US.

**Results:**

We conducted analysis to assess clinical burden and direct medical costs from 44 patient records in CHESS US, and of direct non-medical costs, indirect costs, and humanistic burden (using the EQ-5D-5L) from 57 patients in CHESS US+. The mean annual bleed rate was 1.73 (standard deviation, 1.39); approximately 9% of patients experienced a bleed-related hospitalization during the 12-month study period. Nearly all patients (85%) reported chronic pain, and the mean EQ-5D-5L utility value was 0.76 (0.24). The mean annual direct medical cost was $614,886, driven by factor IX treatment (mean annual cost, $611,971). Subgroup analyses showed mean annual costs of $397,491 and $788,491 for standard and extended half-life factor IX treatment, respectively. The mean annual non-medical direct costs and indirect costs of hemophilia B were $2,371 and $6,931.

**Conclusions:**

This analysis of patient records and patient-reported outcomes from CHESS US and CHESS US+ provides updated information on the considerable clinical, humanistic, and economic burden of hemophilia B in the US. Substantial unmet needs remain to improve patient care with sustainable population health strategies.

## Background

Hemophilia B is a rare X-linked recessive genetic disorder characterized by abnormal bleeding due to defective or missing clotting factor IX (FIX) [1]. Approximately 6000 people in the US have hemophilia B [2], which occurs predominantly in males [3]. The severity of hemophilia B is based on the endogenous FIX level. Mild (FIX activity 5 to 40% IU/dL) and moderate (1 to < 5% IU/dL) hemophilia B are generally associated with excessive bleeding with trauma, and sometimes, spontaneous bleeding [4–7]. In severe disease (FIX level < 1 IU/dL), spontaneous and recurrent bleeding into joints is frequent, leading to deformity, arthritis at an early age, and long-term sequelae [8–11].

Hemophilia B is known to cause substantial functional limitations and reduced health-related quality of life (HRQoL) [12–14] related to bleeding and joint damage, and other disease-related complications [15, 16]. Prophylactic administration of FIX replacement therapy is the standard of care for severe hemophilia B in the US [17–20], and has helped improve life expectancy to approach that of the general population [21]. Extended half-life (EHL) and standard half-life (SHL) formulations are administered often, every 1–2 weeks and 2–3 times per week, respectively, which incurs a treatment burden on patients [22, 23] and a notable economic burden to healthcare systems and society [24–27].

Studies of the nonclinical burden of hemophilia B often focus on the cost of FIX prophylaxis, which accounts for the majority of direct costs, as analyzed in a recent analysis of administrative claims and a systematic literature review [26, 28]. Few recent studies have examined indirect costs and non-medical direct costs, which comprise a smaller proportion of total costs but impose a considerable burden on patients, their caregivers, and society [27, 29]. The European CHESS study reported lost wages for both patients and caregivers, and substantial costs to patients related to the demands of managing both the disease and lifelong treatment [30]. Such estimates of patient-centered burden have been less well characterized in the United States since the advent of newer treatment options, such as EHL prophylaxis. The hemophilia treatment advances that have prolonged life expectancy have also brought attention to examining the quality of those additional life-years, requiring deeper understanding of the impact of disease and treatment on all aspects of patients’ lives. As such, accounting for both direct and indirect costs is important for evidence-based policy and population health management decisions.

The ‘Cost of Severe Hemophilia across the US: a Socioeconomic Survey’ (CHESS US) study was designed to provide population-based insights on the real-world burden of severe hemophilia in the US. We used the CHESS US datasets to further characterize the clinical, humanistic, and economic burden of severe hemophilia B for patients treated with FIX prophylaxis in the US.

## Results

### Patients

Of 576 patients in CHESS US, 132 (23%) had severe hemophilia B of whom 33% (44/132) had an evaluable record of continuous FIX prophylaxis and were included in the analysis (20 received SHL, 24 received EHL). Of the 88 patients with severe haemophilia B who were excluded, 54 had on-demand treatment, 18 had intermittent prophylaxis, 1 had no record of treatment, and 15 with continuous prophylaxis had inadequate treatment information (n = 2) or < 15 IU/kg dose per infusion (n = 13). Of 356 patients in CHESS US+, 97 (27%) had severe hemophilia B of whom 59% (57/97) had a record of FIX prophylaxis and were included in the analysis (22 received SHL, 35 received EHL).

Baseline demographic and clinical characteristics for both analysis cohorts are summarized in Table 1. In CHESS US, patients were on average 28 years old and had a mean weight of 75.7 kg. Patients in CHESS US+ appeared to be slightly older (mean age, 35.6 years) and slightly heavier (mean weight, 85.6 kg). Across cohorts, half of patients reported full-time employment (52% and 49%, respectively), and nearly all patients reported insurance coverage (82% and 100%). The most common medical comorbidities were anxiety, depression, osteoarthritis, and type 2 diabetes. We observed a low prevalence of hepatitis B, hepatitis C, and human immunodeficiency virus (HIV) in both cohorts, consistent with the average age in each cohort.

**Table 1:** Baseline demographics and clinical characteristics

	CHESS US *n* = *44*	CHESS US+ *n* = 57
Age, mean (SD) (years)	27.64 (11.05)	35.84 (12.69)
Weight, mean (SD) (kg)	75.71 (13.42)	85.57 (21.15)
Comorbidities, n (%)		
Anxiety	1 (2.3%)	16 (28.1%)
Depression	3 (6.8%)	13 (22.8%)
Type 2 diabetes mellitus	3 (6.8%)	1 (1.8%)
Hepatitis B	0	8 (14.0%)
Hepatitis C	2 (5%)	0
HIV	0	5 (8.8%)
Osteoarthritis	0	12 (21.1%)
Osteoporosis	0	1 (1.8%)
Employment status, n (%)		
Full-time employed	20 (52.3%)	28 (49.1%)
Part-time employed	4 (9.1%)	14 (24.6%)
Unemployed/Student/Retired	17 (38.6%)	15 (26.3%)
Insurance coverage, n (%)		
Yes	36 (81.8%)	57 (100%)

*HIV* human immunodeficiency virus

### Clinical outcomes

The mean annual bleed rate (ABR) from CHESS US was 1.73 (SD 1.39; median 2.0; Table 2). At least one bleed-related hospitalization was reported by 9.1% of CHESS US patients in the previous year, with a mean length of stay of 0.3 days. One-fifth (18%) of patients reported at least one target joint, and 11% of patients reported at least one problem joint.

**Table 2:** Clinical outcomes

	CHESS US *n* = 44
ABR	
Mean (SD)	1.73 (1.39)
Median (range)	2.00 (0–5)
Bleed-related hospitalizations	
One or more admissions	4 (9.1%)
Inpatient stay, mean (SD)	0.34 (1.22)
ICU stay, mean (SD)	0.23 (0.86)
Target joints	
0 TJ	36 (81.8%)
1 TJ	4 (9.1%)
2+ TJ	4 (9.1%)
Problem joints	
0 PJ	39 (88.6%)
1 PJ	2 (4.5%)
2+ PJ	3 (6.8%)

### Humanistic outcomes

The mean reported EQ-5D-5L score was 0.74 (SD 0.26). More than one-quarter (28%) of patients from CHESS US+ reported chronic pain ratings ≥ 6/10, and half (56%) reported pain 1–5/10 on average over the past year. Slightly more than half of patients (56%) from CHESS US+ reported that their daily lives were compromised by hemophilia B, and nearly all (91%) reported adapting their FIX treatment schedule to account for anticipated physical activity demands (Table 3).

**Table 3:** Humanistic outcomes

	CHESS US+ *n* = 57
EQ_5D_5L utility value	
Mean (SD)	0.74 (0.26)
Chronic pain	
No pain	9 (15.8%)
Pain level 1–5	32 (56.1%)
Pain level 6–10	16 (28.1%)
Daily life compromised by hemophilia	
Yes	32 (56.1%)
No	25 (43.9%)
Adapt treatment regimen (physical activity)	
Yes	52 (91.2%)
No	5 (8.8%)

### Economic outcomes

**Fig. 1:**
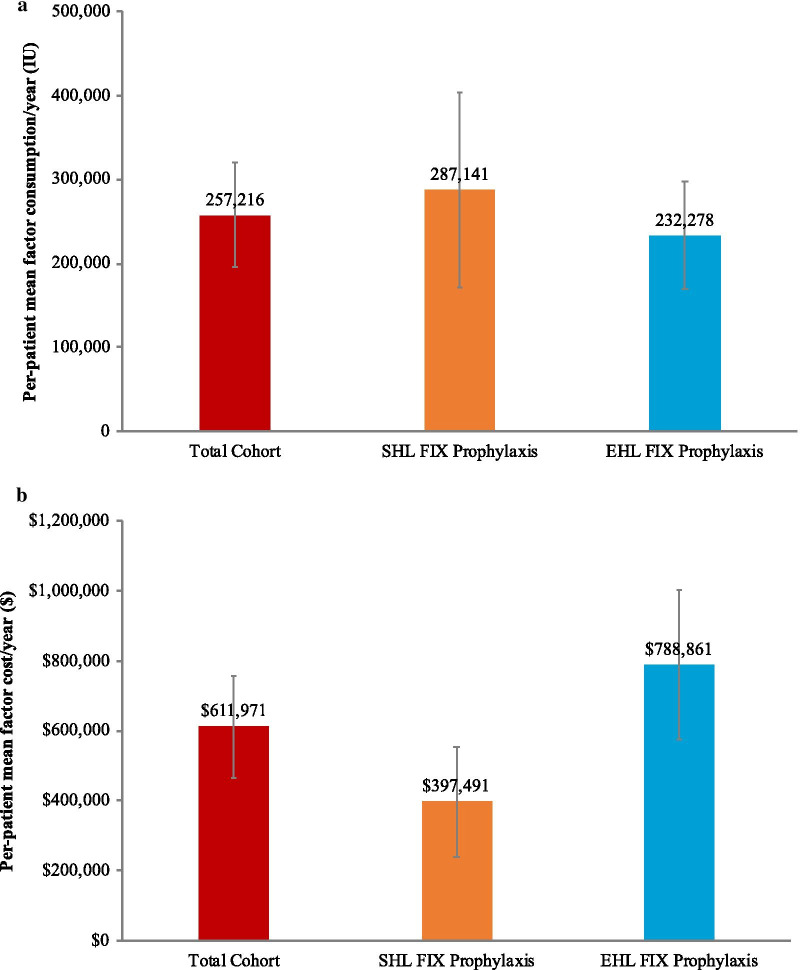
Annual FIX usage (**a**) and costs (**b**). *EHL* extended half-life, *SHL* standard half-life

The annual FIX usage per patient from CHESS US was 257,216 IU (Fig. 1), with an annual cost of $611,971. Usage of FIX among those who received SHL was 287,141 IU, with a corresponding annual cost of $397,491. For patients who received EHL, annual FIX usage was 232,278 IU with a corresponding annual cost of $788,861. Total annual direct medical costs of hemophilia B from CHESS US were $614,886, driven almost entirely by the cost of FIX treatment ($611,971; Table 4). The annual direct medical cost of hemophilia B excluding FIX treatment was $2885.

**Table 4:** Economic outcomes: direct medical costs

Mean (SD), unless noted	CHESS US *n* = *44*
Hemophilia consultation	$271 ($113)
Other consultation	$73 ($66)
Blood tests	$191 ($116)
Other tests	$87 ($268)
Ward inpatient	$2,194 ($7,845)
ICU	$4,123 ($14,284)
Factor IX	$611,971 ($497,281)
Total direct medical cost (N = 44)—excluding FIX	$2,885 ($7,857)
Total direct medical cost (N = 44)—including FIX	$614,886 ($498,839)

Costs were calculated in 2019 USD based on Factor IX consumption reported by the physicians, using Factor IX unit costs from IBM® Micromedex® RED BOOK®

Annual non-medical direct costs from CHESS US+ were $2371, driven primarily by caregiver expenses ($1566 for professional and informal caregiving; Table 5). Annual indirect costs of $6931 were driven by hemophilia-related unemployment and early retirement.

**Table 5:** Economic outcomes: non-medical and indirect costs

Mean (SD), unless noted	CHESS US+ *n* = *57*
Non-medical cost	
Alternative and complimentary therapies	$150 (452)
Devices and home alterations	$28 (75)
Over the counter medications	$178 (323)
Disability entitlement	$116 (343)
Transit	$333 (351)
Professional caregiver	$947 (5,081)
Informal caregiver	$619 (3,179)
Indirect cost	
Absenteeism	$760 (2,286)
Early retirement/unemployed due to hemophilia	$6,171 (16,640)
Total non-medical cost	$2,371 (6,184)
Total indirect cost	$6.931 (16,510)

## Discussion

Despite recent improvements in treatment options, our analysis of the CHESS US and CHESS US+ datasets have shown a persistent and comprehensive clinical, humanistic, and economic burden of hemophilia B on patients receiving FIX prophylaxis, with substantial FIX treatment-driven costs to payers and society. Bleeding-related health resource use was accompanied by chronic pain and compromised employment due to hemophilia, along with non-medical and indirect costs related to self-management and use of professional or informal caregivers. These personal and societal costs were observed in addition to high treatment costs, emphasizing the remaining unmet needs for reducing the burden of hemophilia B with sustainable population health strategies and treatment options.

Despite receiving FIX prophylaxis, patients with severe hemophilia B continue to experience breakthrough bleeding and may eventually develop hemophilic arthropathy [31]. We observed target joints in approximately 20% of patients, and a mean ABR of 1.73 that was consistent with other recent real-world studies [27, 32]. These breakthrough bleeds could be related to repeated FIX trough periods, during which patients are exposed to higher risk of bleeding [33, 34], and the frequent infusions that may compromise treatment adherence and limit the real-world effectiveness of FIX prophylaxis [33, 34]. Taken together, our findings suggest that even with access to prophylaxis, patients with severe hemophilia B remain at considerable risk for arthropathy and long-term damage.

Our findings are consistent with previous reports of the burden hemophilia B and continuous FIX treatment impose on patients and their families [12–14]. Nearly all (85%) patients in CHESS US+ reported some level of chronic pain, which is a patient-important outcome highlighted in the US Food and Drug Administration (FDA) Patient-Focused Drug Development (PFDD) initiative for hemophilia [35]. We observed a corresponding level of humanistic burden, with an average EQ-5D-5L utility value of 0.74 which is consistent with the range of values reported in a recent systematic literature review [36]. More than half of patients reported that hemophilia had compromised their daily lives, and nearly all reported an influence of FIX treatment scheduling on their physical activity.

We also observed a substantial economic burden of hemophilia B and its treatment to US payers, with an annual direct medical cost for severe hemophilia B of $614,886, driven by the cost of FIX replacement therapy ($611,971, or 99% of total direct medical costs). The annual cost of EHL FIX prophylaxis was nearly double that for SHL FIX ($788,861 and $397,491, respectively). Our findings from medical record abstractions (CHESS data analyses) are consistent with recent analyses of administrative claims from large commercial payer databases [26], but provide additional context from physician- and patient-reported clinical and humanistic burden. Similar to our findings, Tortella and colleagues reported 30% lower mean monthly dispensed IUs and 54% higher mean monthly costs for EHL versus SHL regimens among 296 commercially insured US patients with moderate or severe hemophilia B [26]. While this study was not meant to compare EHL versus SHL regimens, it is important to note that assessment of direct costs only did not capture the potential clinical benefits and reduced treatment burden associated with an EHL regimen [37, 38]. In addition to FIX replacement therapy, the costs of medical encounters such as hospitalizations (and intensive care unit admissions) and physician office visits represent additional financial burden to the healthcare system.

Our study also quantified the direct non-medical and indirect costs associated with hemophilia B and FIX replacement therapy. Direct non-medical costs were mainly driven by caregiver expenses, both professional and informal, and indirect costs were comprised largely of hemophilia-related unemployment and early retirement. Findings were consistent with those of the prospective, longitudinal Hemophilia Utilization Group Studies Part Vb (HUGS Vb) study of patients with hemophilia B from 10 HTCs in the US [27]. The direct non-medical and indirect costs associated with hemophilia B may comprise a relatively small proportion of the total cost, but nonetheless represent a significant burden to patients, employers, and society in the form of lost income and productivity for both patients and caregivers [27, 39].

These findings should be interpreted in the context of certain strengths and limitations. While we were able to include patient-reported outcomes, any retrospective, cross-sectional study is subject to certain limitations. Both the chart review and patient survey could be prone to selection bias, recall bias, and/or potential errors in data abstraction. We used data from two distinct cohorts for clinical and economic burden vs humanistic, and reported the results separately. Clinical burden and direct medical costs were based on physician-reported data in the CHESS US cohort, which may not have reflected the exact costs incurred by the ultimate payer. Since this analysis focused on patients without inhibitors receiving consistent prophylaxis treatment, our findings are not generalizable to populations with inhibitors and those receiving on-demand treatment or periodic prophylaxis. Other unmeasured variables may also limit generalizability of these findings to specific types of patients with hemophilia B in the US. Nonetheless, our findings were consistent with reports from other data sources, and suggest that the CHESS US and CHESS US+ datasets are well suited to provide insights into the burden of hemophilia to patients and society in the US.

## Conclusions

We observed a substantial clinical and humanistic burden of hemophilia B on patients receiving FIX prophylaxis in the US, and substantial FIX treatment-driven costs to the society. The CHESS US and CHESS US+ cohorts provided important insights for personalized patient care and population health management. Despite recent therapeutic advances in the treatment of severe hemophilia B, tangible unmet needs remain to better serve patients with this lifelong condition in a sustainable manner.

## Methods

### Study design

Following a similar design and methodology as the original CHESS study in Europe [30], CHESS US is a cross-sectional database of information extracted from medical record notes and physician-reported information, by hematologists and hemophilia care providers for adults with severe hemophilia A or B in the US. Patient demographics, symptoms, treatment information and healthcare resource use for 576 patients was abstracted by 100 hemophilia care providers into a patient record form (PRF). Providers were recruited between 2017 and 2018. Each provider completed a PRF form with at least 12 months of retrospective data from each patient [30].

To supplement the CHESS US database, CHESS US+ is a separate, cross-sectional database of patient-reported information and outcomes provided by adult patients with hemophilia A or B using a patient panel approach. Participants used a secure web-based platform to complete a patient self-completion (PSC) form and respond to patient-reported outcome measures. The questionnaire collected information on out-of-pocket expenditures for transportation, alternative and complimentary therapy (i.e., physical rehabilitation), and impact on productivity and attendance at work. All forms were completed in 2019 and captured a retrospective period of 12 months.

The CHESS US and CHESS US+ sample populations were drawn in approximate proportions to those with hemophilia A or B in the US population [40]. This study was governed and approved by the University of Chester Ethics Committee in partnership with the National Hemophilia Foundation (NHF). All data were pseudonymized.

### Study population

The population for this analysis included patients from CHESS US and CHESS US+ with severe hemophilia B and no medical history of FIX inhibitors who were treated with continuous FIX prophylaxis. We excluded patients treated with on-demand therapy, patients without information on type of FIX treatment (SHL or EHL), patients who received intermittent prophylaxis, and patients whose prophylactic dose was below 15 IU/kg (which was likely associated with data entry errors).

### Study outcomes and analyses

Clinical outcomes included ABR, bleed-related hospitalizations, and joint health from CHESS US, and patient-reported chronic pain from CHESS US+. Joint health was captured using both ‘target joints’ based on the International Society on Thrombosis and Haemostasis (ISTH) definition [41] and ‘problem joints’ based on a clinician and patient consensus definition on chronically damage joints [42]. In this case, a ‘problem joint’ was defined as any joint that has been permanently damaged as a result of a bleeding disorder, with or without persistent bleeding, and may involve chronic pain and/or limited range of movement due to compromised joint integrity such as chronic synovitis and/or hemophilic arthropathy [42]. Patients reported chronic pain over the last 12 months on a scale of 0–10 where 0 indicated “no pain” and 10 indicated “extreme pain.” Comorbidities were reported by physicians based on review of the medical chart for CHESS US, and by patient-reported responses regarding current diagnoses in CHESS US+ (“Are you currently diagnosed with any of the following conditions? [Check all that apply]”).

Humanistic outcomes from CHESS US+ included HRQoL and the impact of hemophilia B on daily life and physical activity. HRQoL was measured using the EuroQoL 5-Dimension 5-Level (EQ-5D-5L) with utility values derived from the recently published US value set [43].

Economic outcomes included FIX usage and direct medical costs from CHESS US, and non-medical and indirect costs from CHESS US+. All costs were reported in 2019 USD ($). The annual cost of FIX treatment was calculated based on FIX usage from CHESS US and FIX unit costs from IBM® Micromedex® RED BOOK® [44]. Direct medical costs included FIX consumption, consultations with hemophilia specialists and the multidisciplinary team, testing, hospital stay and ICU use. Non-medical costs included expenses such as use of professional caregiving, travelling to the Hemophilia Treatment Center (HTC), alternative and complimentary therapies, devices and home alterations, over the counter medications, transfer payments, professional and informal caregiving, and transit costs. Indirect costs comprised the cost associated with absenteeism, presenteeism, and early retirement/forced unemployment due to disease burden, and were valued using the human capital approach. Productivity costs (losses) estimated the value of lost time from work due to haemophilia-related absenteeism and long-term disability using patient-reported work impairment and mean reported earnings at a national level.

All outcomes were summarized using descriptive statistics. Results are presented as means with standard deviations for continuous variables or as number and proportion of patients for categorical variables. All analyses were conducted using Stata 16.

## Data Availability

The data that support the findings of this study may be available from HCD Economics, Ltd but restrictions apply to the availability of these data, which were used under license for the current study, and so are not publicly available. Data may be available from the authors upon reasonable request and with permission of HCD Economics Ltd.
